# Latent profiles of academic delay of gratification and the mediating role of academic self-efficacy in the pathway to academic involution among Chinese college students

**DOI:** 10.3389/fpsyg.2026.1851024

**Published:** 2026-05-18

**Authors:** Xiaoli Ye, Tingting Cheng, Wei Yang, Jingjing Zhou

**Affiliations:** 1 Institute of Higher Education, Anhui University, Hefei, China; 2 School of Education, South-Central Minzu University, Wuhan, China

**Keywords:** academic delay of gratification, academic involution, academic self-efficacy, Chinese college student’s, latent profile analysis, mediation analysis

## Abstract

**Introduction:**

Against the backdrop of increasingly intense educational competition, college students have generally fallen into the dilemma of academic involution. This study integrates variable-centered and person-centered analytical approaches to explore the latent categorical characteristics of academic delay of gratification (ADOG) among Chinese college students, and to further examine the internal mechanisms linking ADOG, academic self-efficacy, and academic involution.

**Methods:**

Between June and August 2025, this study employed a convenience sampling method to distribute online questionnaires to Chinese college students. All students who completed valid questionnaires were included in the analysis. A total of 717 Chinese college students participated in this study, consisting of 284 males (39.6%) and 433 females (60.4%), with a roughly even distribution across the first to fourth academic years. Data were collected using the Academic Delay of Gratification Scale, the Academic Self-Efficacy Scale, and the Academic Involution Scale. Latent profile analysis (LPA) was conducted to classify the latent profiles of ADOG, and correlation analysis, mediation analysis, and moderated mediation modeling were performed to examine the relationships among the variables.

**Results:**

The results showed that ADOG among college students could be classified into three latent profiles: low-level, medium-level, and high-level groups. These three groups demonstrated significant differences in levels of academic self-efficacy and academic involution. Variable-centered mediation analysis revealed that academic self-efficacy partially mediated the relationship between ADOG and academic involution. In the person-centered analysis, using the low-level group as the reference, academic self-efficacy exerted a partial mediating effect in the association between high-level ADOG and academic involution.

**Discussion:**

This study confirms the group heterogeneity of ADOG among college students. It also clarifies the internal mechanisms linking the three variables from both variable-centered and person-centered perspectives. The findings provide solid empirical evidence and practical guidance for universities to formulate differentiated intervention strategies for academic pressure and to guide college students in responding rationally to academic competition.

## 1 Introduction

Against the dual backdrop of the massification of higher education and intensifying social competition, Chinese university students are experiencing unprecedented academic and job pressure (Zheng W., 2024). To compete for scarce high-quality educational resources, students unconsciously find themselves caught up in intense competition. The term academic “involution” has accordingly become a key concept for describing this phenomenon and has gradually emerged as an important topic in research on Chinese higher education (Kang and Jin, 2020). The term involution can be traced back to 1963, when anthropologist Geertz, studying agricultural civilizations, defined it as a phenomenon whereby a cultural pattern, after reaching a seemingly fixed form, neither stabilizes nor evolves into a new pattern, but instead sustains itself by continuously increasing its internal complexity (Geertz, 1963). With the shift of social context, the meaning of involution has gradually expanded. In the field of education, it is often used to refer to excessive competition (Liang, 2023), a usage reflecting the anxiety embedded in a high-pressure competitive culture.

In the current context of Chinese higher education, academic involution among university students has become remarkably widespread. According to the China College Student Academic Development Report (2024), 68.3% of students at elite universities suffer from persistent fatigue, and 52.7% experience a decline in achievement motivation, with “involution pressure” identified as the primary cause. These figures indicate that academic involution has become a prominent group-level phenomenon that cannot be overlooked. Therefore, exploring effective ways to alleviate this trend among university students carries considerable theoretical value and practical significance.

Notably, although academic involution is a concept rooted in China’s domestic educational context, the excessive academic competition it describes is not unique to Chinese students. Rather, it is a global phenomenon that is particularly prominent and highly consistent within the East Asian Confucian cultural sphere. In China, under the influence of traditional cultural values that emphasize academic achievement and the modern examination-oriented education system, schools typically evaluate students primarily based on their academic performance (Liang, 2023). Against this backdrop, to maintain a competitive advantage, college students are more likely to increase their learning investment without an upper limit, ultimately manifesting as academic involution (Si, 2022). Similarly, South Korea regards the college entrance examination as a core social screening mechanism, which has given rise to an “education fever” and a highly competitive educational environment (Kim and Bang, 2017; Jarvis et al., 2020). Surveys indicate that South Korean students spend approximately twice as much time studying as their American peers and participate more frequently in private tutoring after school and on weekends (Lee and Larson, 2000; Huan et al., 2008). In Japan, students engage in shadow education (out-of-school tutoring) from an early age (Samuell, 2024), and “juku” (cram schools) as well as “preparatory schools” have become almost a standard fixture for middle-class families. In contrast, educational competition in Western countries manifests more as implicit screening based on class-based resource disparities, such as the costly investment required to cultivate “comprehensive competencies” in U. S. elite education (Chetty et al., 2026). Therefore, academic involution is not only a structural dilemma in China’s period of social transformation but also a cross-cultural collective anxiety about “academic success” in the context of a worldwide scarcity of high-quality educational resources.

Competition is not inherently negative; rather, it is a double-edged sword. Moderate academic competition can stimulate learning motivation and creativity, and promote cooperative interaction among peers (Zhao, 2016). However, when competition degenerates into involution, its negative effects far outweigh the potential benefits, necessitating a clear distinction between academic involution and normal academic engagement. Unlike productive academic engagement, academic involution is characterized by excessive effort driven by rank maintenance under resource scarcity, featuring increasing marginal costs and diminishing marginal returns, and ultimately degenerating into unproductive consumption. Based on this distinction, the negative impacts of academic involution manifest in two main aspects. First, constrained by a rigid evaluation system, students devote substantial energy to standardized examinations and fall into extreme competition over test scores (Liang, 2023; Si, 2022). Second, long-term exposure to involution tends to induce psychological problems such as anxiety and burnout, and even weakens students’ intrinsic recognition of the value of learning (Chen et al., 2021).

Academic delay of gratification (ADOG) is the tendency to voluntarily forgo immediate rewards for long-term academic goals (Mischel et al., 1988). Extant research has confirmed that ADOG is significantly associated with academic engagement and has positive effects on academic development (Zhou et al., 2022; Chen and Yeung, 2025). However, the effects of ADOG are not uniformly positive. As a core manifestation of self-control and self-regulation (Jiao et al., 2022), excessively high ADOG may trigger over-control and irrational investment, driving students to engage in academic involution, either passively or actively (Liu et al., 2022; Ren et al., 2022). Meanwhile, students with a high level of ADOG generally report stronger academic self-efficacy (Bembenutty and Karabenick, 1998). As positive psychological resources, ADOG and academic self-efficacy can jointly enhance academic engagement (Dogan, 2015). Nevertheless, the existing literature has not systematically examined how ADOG, academic self-efficacy, and academic involution covary, either theoretically or empirically.

Moreover, the majority of earlier studies have adhered to a variable-centered research perspective, treating variables such as ADOG as continuous and homogeneous entities and ignoring the potential variability and differences among individuals. To address the aforementioned research gaps, Latent Profile Analysis (LPA) will be adopted in this study to identify the latent profile characteristics of college students’ ADOG, with a focus on examining the differences in academic involution and academic self-efficacy across various profile groups. Additionally, to comprehensively reveal the internal mechanism linking ADOG, academic involution, and academic self-efficacy, this study will further examine the mediating role of academic self-efficacy in the relationship between ADOG and academic involution within different latent profiles. This research provides important empirical insights for educators to understand the heterogeneity of ADOG and offers theoretical backing and practical approaches to alleviate academic involution and promote healthy academic development among college students.

## 2 Literature review and theoretical hypotheses

### 2.1 Academic delay of gratification and academic involution

Research indicates that students with higher levels of ADOG demonstrate greater proficiency in deploying cognitive strategies and resource management strategies, maintain higher levels of learning engagement, and exhibit stronger resilience and perseverance when facing academic challenges (Bembenutty, 2022; Zhou et al., 2026). Against a global context, an increasing number of studies have expanded understanding of the functions of ADOG and the generalizability of its findings. For instance, Datu et al. (2020) examined predictors of ADOG in the Philippine context, focusing on how collectivistic cultural contexts foster ADOG. Yanaoka et al. (2022) administered delay-of-gratification tasks to young children in the United States and Japan to investigate how reward types (i.e., marshmallows vs. gifts) influence delay behavior across contexts. Their results revealed cultural differences in habits and values related to waiting, which may contribute to variations in delay ability across groups. Notably, Generation Z students, due to excessive social media use and exposure to various instant-gratification distractions, commonly develop a preference for immediate rewards. In response, Chakraborty and Chechi (2026) noted that as a key variable in self-regulated learning, ADOG can effectively counteract the negative effects of such immediate-gratification tendencies and redirect students toward the pursuit of long-term academic goals.

The term “involution” was initially used to describe constraints on cultural development (Goldenweiser, 1913). Later, Geertz (1963) defined it as a developmental pattern in which a system becomes increasingly refined and complex internally without achieving qualitative transformation. Today, the concept is widely employed in China to characterize excessive competition in higher education, and a strong atmosphere of academic involution has emerged among Chinese university students (Zhou et al., 2022). Unlike ordinary academic engagement, academic involution essentially represents excessive learning investment driven by competitive motives. Its core feature is the continuous increase in students’ time and effort input without a corresponding improvement in learning efficiency or innovative capacity, ultimately resulting in internally consumptive competition (Zheng et al., 2022). Under intense pressure related to academic advancement and employment, university students commonly engage in intensive learning efforts to pursue long-term goals (Ren et al., 2022). Students with high academic delay of gratification often invest greater effort to avoid procrastination and maintain competitive advantages, a tendency that further fuels academic involution (Poon et al., 2021). Accordingly, it can be inferred that university students with higher levels of ADOG tend to devote more time and energy to academic activities due to their stronger self-control. In the current highly competitive educational context, increased academic investment ultimately exacerbates academic involution. Based on the above discussion, ADOG is positively associated with the level of academic involution among university students. Therefore, we propose the following hypothesis:

*H1*: ADOG significantly and positively predicts academic involution among college students.

### 2.2 Academic delay of gratification and academic self-efficacy

Academic self-efficacy stems from Bandura’s social cognitive theory and refers to students’ subjective judgment of their ability and confidence to complete academic tasks and achieve learning goals (Zheng C., 2024). Research suggests that academic self-efficacy essentially reflects students’ confidence in academic settings; it is not only a key factor influencing academic performance (Cascio et al., 2013; Taipjutorus et al., 2012) but also an important indicator of mental health. Furthermore, as a core mechanism of self-monitoring and a key motivational variable within self-regulated learning frameworks, academic self-efficacy has been consistently confirmed to be closely associated with students’ learning attitudes and behaviors (Honicke and Broadbent, 2016).

The positive association between ADOG and academic self-efficacy has been widely validated. To accurately measure delay of gratification tendencies in academic contexts, Bembenutty and Karabenick (1998) developed the first dedicated Academic Delay of Gratification Scale. Their results showed that students with higher delay of gratification tendencies had significantly higher self-efficacy ratings and stronger intrinsic motivation, providing initial empirical support for the association between the two constructs. Subsequent longitudinal studies have further substantiated this conclusion. Beymer et al. (2023) conducted a one-semester longitudinal study among higher education students and found that those with higher initial levels of delay of gratification exhibited significantly higher academic self-efficacy at the end of the semester compared to those with lower initial levels. Moreover, this positive association was more pronounced for academic tasks requiring long-term commitment. Additionally, research has indicated a direct link between academic delay of gratification and students’ confidence and effort in persisting with academic tasks (Bembenutty, 1999). The underlying mechanism of this positive association can be understood from two perspectives. First, a high capacity for delay of gratification enables students to maintain sustained engagement when facing academic difficulties or temptations. This sense of control over one’s learning behavior is itself an important component of self-efficacy (Bembenutty, 2011; Pintrich et al., 1993). Second, sustained engagement of students with high delay of gratification often leads to better academic performance, and favorable academic outcomes, in turn, validate students’ perceptions of their own abilities, further enhancing their academic self-efficacy (Schunk et al., 2008). Based on the above analysis, we propose Hypothesis 2:

*H2*: ADOG significantly and positively predicts college students' academic self-efficacy.

### 2.3 Individual differences in ADOG

Previous studies on ADOG have predominantly employed variable-centered approaches. These approaches conceptualize ADOG as a continuous and homogeneous entity, primarily examining its linear relationships with academic performance and other related variables. However, from the perspective of self-regulated learning theory (Zimmerman, 2000), learners’ self-regulation is a complex process involving cognitive, motivational, and behavioral components. Different individuals may exhibit distinct self-regulation patterns due to variations in their intrinsic motivation, task value appraisal, and self-efficacy beliefs. For instance, Vanslambrouck et al. (2019) revealed that students’ self-regulated learning patterns can be characterized by three distinct profiles: high, moderate, and low. Therefore, as a specific form of self-regulation, ADOG among college students may possess inherent heterogeneity, manifesting as latent subtypes composed of different behavioral patterns and psychological characteristics. Variable-centered approaches are ill-suited to reveal the potential heterogeneity of individuals across different variable combinations (Lin S. et al., 2023). In contrast, person-centered latent profile analysis (LPA) can identify latent subgroups characterized by distinct profiles based on individuals’ response patterns on ADOG-related indicators (Dai et al., 2022). This method helps to identify typical performance types of college students in ADOG, thereby providing a basis for subsequent precise interventions targeting different groups.

Existing research has attempted to classify ADOG, for instance, Zu et al. (2021) found through interviews that there are three levels of ADOG, including high, medium, and low, and learners, teachers, and parents should intervene based on the different levels of ADOG of learners to promote their ADOG. Meanwhile, in constructs closely related to ADOG, LPA studies have commonly identified three typical latent classes. For example, Zhou et al. (2024) found that nursing students’ academic procrastination exhibited low, moderate, and high levels. Cano et al. (2024) classified university students’ academic engagement into three types: burned-out, moderately engaged, and engaged. Moreover, although evidence from existing research confirms that students with high ADOG are inclined to use learning strategies and exhibit stronger autonomous learning abilities (Bembenutty and Karabenick, 2004), it is still unclear whether there are differences in academic self-efficacy and academic involution at different levels of ADOG among college students. Given this, this study will identify the latent profiles of ADOG among college students through latent profile analysis and further examine the differences in academic self-efficacy and academic involution among different latent profiles to more deeply reveal the differentiated paths through which ADOG affects academic performance. We make the following hypothesis:

*H3*: There are three latent profiles of ADOG among college students, which are high ADOG, medium ADOG, and low ADOG. Groups with different levels of ADOG have significant differences in academic self-efficacy and academic involution. The latent profile group with a higher level of ADOG has higher academic self-efficacy and academic involution.

### 2.4 The mediation of academic self-efficacy

Social Cognitive Theory (SCT) explains how self-influence and self-regulatory mechanisms activate and control human behavior (Bandura, 1977). Its core concept, triadic reciprocality, emphasizes the dynamic and bidirectional interactions among individual behavior, personal cognitive factors, and the external environment (Bandura, 1991). Furthermore, Bandura’s self-regulation theory posits that individuals are not passive recipients of environmental stimuli; rather, they actively direct their behavior through goal setting, impulse control, self-monitoring, and self-reflection (Bandura, 1991). Drawing on this integrated framework, the present study employs triadic reciprocality to explain the dynamic relationships among the individual, behavior, and environment, while utilizing self-regulation theory to elucidate the intrinsic regulatory mechanisms of ADOG.

ADOG is not merely a time preference but rather a typical self-regulatory strategy. According to self-regulation theory, individuals can actively suppress the impulse for immediate gratification and allocate limited psychological resources toward long-term academic goals (Funder et al., 1983). When this process of self-control and goal persistence leads to goal attainment, it results in mastery experiences, which in turn significantly enhance academic self-efficacy and foster a more positive appraisal of one’s own learning abilities. Second, the regulatory effect of self-efficacy on behavior is context-dependent, reflecting the dynamic cycle of triadic reciprocality. In low-competition or normative academic environments, high self-efficacy promotes appropriate levels of academic engagement. In highly competitive academic environments, however, high self-efficacy may paradoxically trigger excessive self-regulation. Existing research indicates that students with low ADOG and low academic self-efficacy tend to exert less effort and are more prone to academic procrastination (Nemtcan et al., 2022; Özbay et al., 2025). In contrast, individuals with high self-efficacy tend to set more stringent performance standards and engage in continuous self-monitoring (Zimmerman, 1990), ultimately manifesting as high-intensity, repetitive over-engagement (Van Dinther et al., 2011).

This over-engagement, driven by high self-efficacy, subsequently reshapes the external academic environment. As individuals’ efforts escalate continuously, they converge into a collective effect that raises the benchmark of overall academic competition. The heightened competitive standards, reshaped by individual behaviors, then serve as new external pressures, prompting students to further intensify their self-regulation. This recurring cycle of environmental pressure, behavioral adaptation, and escalating standards constitutes the core mechanism underlying the formation of academic involution.

A substantial body of empirical research supports the positive association between academic self-efficacy and academic engagement (Dogan, 2015; Majer, 2009). Defined as an individual’s belief in their capacity to successfully complete academic tasks, academic self-efficacy directly influences their motivation and learning engagement (Thijs and Verkuyten, 2008). Specifically, students who are confident in their ability to complete academic tasks tend to engage more actively in those tasks. Conversely, those who lack such confidence reduce the time and effort they invest (Meng and Zhang, 2023). Researchers have further noted that high academic self-efficacy leads students to form positive expectations about achieving their academic goals, and this confidence drives them to actively invest greater time and effort in learning (Schunk and Mullen, 2012).

Based on social cognitive theory, self-regulation theory, and relevant empirical evidence, this study proposes that ADOG influences academic involution through the mediating role of academic self-efficacy. Specifically, high ADOG helps individuals accumulate mastery experiences and enhance self-efficacy. In a highly competitive academic context, high self-efficacy motivates individuals to set higher standards and maintain persistent engagement, ultimately leading to academic involution. Notably, most existing studies treat ADOG as a continuous variable and rarely adopt a person-centered approach (e.g., latent profile analysis) to examine the mediating mechanisms across different subgroups. Whether academic self-efficacy mediates the relationship between distinct ADOG profiles and academic involution, and whether such effects demonstrate group heterogeneity, remains to be systematically tested. Accordingly, this study proposes the following hypothesis:

*H4*: Academic self-efficacy mediates the relationship between ADOG and academic involution among college students. Specifically, compared with profile groups characterized by lower levels of ADOG, those with higher levels of ADOG exhibit a stronger positive indirect association between ADOG and academic involution through academic self-efficacy.

### 2.5 The current study

This study focuses on Chinese college students to explore the mechanism linking ADOG to academic involution, with particular emphasis on the mediating role of academic self-efficacy. Given that variable-centered approaches overlook the heterogeneity of ADOG and fail to adequately capture the characteristics and effects of different ADOG subtypes, this study adopts a mixed design combining variable-centered and person-centered methods. It systematically identifies potential ADOG subtypes among college students, compares the differential patterns of academic self-efficacy and academic involution across these subtypes, and clarifies how the associations with the mediating and outcome variables vary by ADOG profile. Based on these findings, the study proposes differentiated intervention strategies tailored to the core characteristics and potential risks of each profile, thereby offering more actionable solutions for academic psychological counseling in higher education institutions.

## 3 Methods

### 3.1 Participants

This study employed convenience sampling to recruit college students from three universities in Anhui and Hubei provinces, China. Data were collected via a questionnaire survey, which comprised four sections: demographic information, ADOG, academic self-efficacy, and academic involution. The survey was conducted from June to August 2025 using Questionnaire Star, a widely adopted professional questionnaire platform in China. An anonymous approach was adopted throughout to protect participants’ privacy. All participants accessed the questionnaire through a uniform electronic link and completed it at their convenience. The study strictly adhered to academic research ethics. Participation was entirely voluntary and anonymous, and respondents could withdraw at any stage without penalty. Before starting, all participants viewed an informed consent form and were required to confirm their agreement before proceeding. We also assured participants that all data would be used solely for this academic research and would not be disclosed for any other purposes, thereby ensuring information security.

A total of 807 questionnaires were collected from college students. Prior to data analysis, the research team followed predefined data cleaning procedures. Questionnaires with abnormally short completion times, input errors, or missing data were first eliminated. Next, items with non-random missing values or excessively high option repetition rates were removed. Both cases were classified as invalid data. After screening, 717 valid samples were obtained, yielding an effective response rate of 88.8%. The entire data processing strictly adhered to confidentiality principles to protect participants’ personal information. The demographic characteristics of the respondents are presented in Table 1. Specifically, the sample consisted of 39.6% male and 60.4% female students; 83.5% were undergraduates and 16.5% were postgraduates; 52.6% were from the humanities and social sciences, and 47.4% from the natural sciences.

**Table 1:** Sample demographics (*N* = 717).

Category	Number (%)
Gender	
Male	284 (39.6%)
Female	433 (60.4%)
Education level	
Undergraduate student	599 (83.5%)
Postgraduate student	118 (16.5%)
Majors	
Humanities and social sciences	377 (52.6%)
Natural sciences	340 (47.4%)

The study was conducted in accordance with the Declaration of Helsinki and approved by the Institutional Review Board of Anhui University (Approval code: AHUIHE20250525). All participants provided informed consent before completing the questionnaire. Data were anonymized and stored securely.

### 3.2 Measurement

#### 3.2.1 Academic delay of gratification

This study adopted the College Students’ Academic Delay of Gratification Scale (ADOGS), revised by King and Du (2011), which was originally developed by Bembenutty and Karabenick (1998). After being adapted to the Chinese context, it better suits the learning scenarios of Chinese students and meets the measurement requirements of this study. The scale consists of 10 items, covering two dimensions: “classroom ADOG” and “after-class ADOG.” All items present dilemma situations and are scored using a 4-point Likert scale (1 = definitely choose A, 2 = probably choose A, 3 = probably choose B, 4 = definitely choose B). The total score of the scale is positively correlated with college students’ ADOG tendency, and higher scores indicate stronger tendencies. The outcomes of the confirmatory factor analysis revealed that *X*^2^/df = 5.202, NFI = 0.935, TLI = 0.925, CFI = 0.947, RMSEA = 0.077. The Cronbach’s *α* coefficient of the scale was 0.866. The reliability and validity indicators met the academic research standards, indicating its suitability for measuring the sample in this study.

#### 3.2.2 Academic self-efficacy

To assess the academic self-efficacy of college students, this study utilized the revised Chinese version of the Motivated Strategies for Learning Questionnaire (MSLQ-RCV) (Lee et al., 2010), which is designed to investigate students’ beliefs about learning motivation in the Chinese educational setting. Consisting of 7 items, the scale adopts a 5-point Likert scoring method for each item, with 1 indicating “completely disagree” and 5 denoting “completely agree.” The total score of the scale is positively correlated with students’ academic self-efficacy. As shown by the confirmatory factor analysis results, the indicators were as follows: *X*^2^/df = 4.826, NFI = 0.965, TLI = 0.959, CFI = 0.972, and RMSEA = 0.073. The Cronbach’s *α* coefficient of this scale was 0.914. Overall, the scale met the standard requirements of academic research, indicating its suitability for measuring the academic self-efficacy of college students in this study.

#### 3.2.3 Academic involution

The Academic Involution Scale for College Students in China (AISCSC), developed by Wang et al. (2024), was employed in this study to measure the academic involution behaviors of Chinese college students. The scale was specifically designed for Chinese university students and has been demonstrated to have good reliability and validity within the Chinese context. The scale items accurately capture the unique manifestation of academic involution within China’s highly competitive educational environment, characterized by passive academic overinvestment (e.g., “I get up early and come back late every day to study so as not to be left behind”), sacrificial activity participation (e.g., “Although I don’t like it very much, I will attend various lectures so that my comprehensive evaluation results will not be left behind by others”), and instrumental social interaction (e.g., “I will actively interact with teachers and strive to achieve no lower grades than others”). These items are conceptually distinct from academic engagement, which is driven by intrinsic interest and mastery goals. This scale comprises 16 items, split into three dimensions: academic behavior (7 items), social activities (5 items), and social interaction (4 items). Each item was scored using a 5-point Likert scale, with a score range of 1 to 5. A higher overall score on the scale reflects a more intense degree of academic involution among the surveyed college students. The Cronbach’s α coefficients of the three dimensions were 0.858, 0.928, and 0.884. Confirmatory factor analysis yielded fit indices: *X*^2^/df = 4.905, NFI = 0.935, TLI = 0.937, CFI = 0.947, and RMSEA = 0.074. These findings confirm the scale’s good reliability and validity.

### 3.3 Data analysis

All study data were analyzed with SPSS 27.0 and Mplus 8.3. The specific process is as follows: First, conducting descriptive statistics and Pearson correlation analysis on all variables. Second, latent profile analysis (LPA) was carried out at the item level to identify different profiles of ADOG with distinct clustering patterns. When conducting LPA with Mplus 8.3, models from 1 to 5 were evaluated successively, and the best classification was identified through the comparison of fit indices. A high-quality classification model should meet the following conditions: (1) lower values of Akaike Information Criterion (AIC), Bayesian Information Criterion (BIC), and sample size-adjusted BIC (ABIC); (2) significant *p*-values for the Lo–Mendell–Rubin likelihood test (LMR) and the adjusted Bootstrapped Likelihood Ratio Test (BLRT); (3) higher entropy values closer to 1. Third, after determining the optimal classification through LPA, univariate ANOVAs and post-hoc tests were used to compare differences in academic self-efficacy and academic involution among different ADOG profiles. Fourth, SPSS PROCESS v4.0 (Model 4) was employed to test the simple and relative mediating effects of academic self-efficacy between ADOG and academic involution.

To reduce the potential common method bias resulting from the self-reporting method of the subjects, this study evaluated the degree of bias by examining common method variance. The Harman single-factor test results showed that the variance explained by the first common factor was 33.17%, which was below the critical value of 40%. Furthermore, this study employed the Unmeasured Latent Method Construct (ULMC) approach to test for common method bias. The results showed that after introducing the common method factor, the CFI increased by 0.023 and the TLI increased by 0.013, both well below the threshold of 0.1. The RMSEA decreased by 0.003 and the SRMR decreased by 0.004, both far below the criterion of 0.05, indicating that the model fit did not improve significantly after the inclusion of the method factor. These findings indicate that there was no significant common method bias in this study.

## 4 Results

### 4.1 Descriptive statistics and correlation analysis

Descriptive analysis results indicated that the mean values of the research variables were ADOG (*M* = 3.01), academic involution (*M* = 3.25), and academic self-efficacy (*M* = 3.57) respectively. The means of ADOG and academic involution among college students fall within the range of 2.50–3.50, indicating a moderate level. This suggests that college students generally possess a certain degree of self-control while exhibiting a relatively high tendency toward “involution.” The mean of academic self-efficacy ranges from 3.50 to 4.50, representing a moderately strong level, indicating that college students’ academic self-efficacy is relatively ideal, with strong confidence in completing their academic tasks. The correlation analysis reveals that all variables are significantly correlated. Specifically, ADOG shows a significant positive correlation with academic involution (*r* = 0.34, *p* < 0.01) and with academic self-efficacy (*r* = 0.26, *p* < 0.01); academic involution also demonstrates a significant positive correlation with academic self-efficacy (*r* = 0.52, *p* < 0.01). The correlation analysis results provide preliminary evidence for the research hypotheses.

### 4.2 LPA model fit and grouping

To explore the latent profile types of college students’ ADOG, starting from one classification, the number of profiles was gradually increased, and the model fit index parameters were compared until the model with the best fit was determined. The main model fit indices evaluated in this study are LL, AIC, BIC, ABIC, Entropy, LMR, and BLRT. For LL, AIC, BIC, and ABIC, smaller values correspond to better model fit; the Entropy value, which is used to judge the classification accuracy, is generally required to be above 0.80. The closer this value is to 1, the higher the accuracy of the classification; when the *p*-values of the likelihood ratio test indices LMR and BLRT are significant (*p* < 0.05), the k-profile model is deemed better than the k-1-profile model. Additionally, if the proportion of a certain profile is less than 5%, it suggests that the classification is not meaningful (Wen et al., 2023). The model fitting results are displayed in Table 2.

**Table 2:** LPA model comparison with different numbers of profiles.

Indicators	Unconditional model				
	1-Class	2-Class	3-Class	4-Class	5-Class
Fit statistics					
LL	−9952.37	−9066.18	**−8714.75**	−8563.13	−8463.43
AIC	19944.74	18194.36	**17513.51**	17232.27	17054.87
BIC	20036.24	18336.18	**17705.66**	17474.75	17347.67
ABIC	19972.74	18237.75	**17572.30**	17306.46	17144.45
Entropy	–	0.85	**0.86**	0.90	0.88
BLRT(p)	–	<0.001	**<0.001**	<0.001	<0.001
LMR(p)	–	<0.001	**<0.05**	<0.05	>0.05
Group size (%)					
C1	717 (100%)	304 (42.40%)	**140 (19.53%)**	88 (12.27%)	79 (11.02%)
C2	–	413 (57.60%)	**313 (43.65%)**	66 (9.21%)	68 (9.48%)
C3	–	–	**264 (36.82%)**	288 (40.17%)	65 (9.07%)
C4	–	–	**–**	275 (38.35%)	227 (31.66%)
C5	–	–	**–**	–	278 (38.77%)

LPA, latent profile analysis; AIC, Akaike information criterion; BIC, Bayesian information criterion; ABIC, adjusted Bayesian information criterion; LMR, Lo Mendell Rubin; BLRT, Bootstrap likelihood ratio test. Indices of the best-fitting model are in boldface.

As the number of profiles increased, LL, AIC, BIC, and aBIC continued to decrease. When the number of profiles reached five, the p-value of the LMR test was no longer significant (*p* > 0.05), indicating that the five-profile model did not significantly improve upon the four-profile model; therefore, it was excluded. We further compared the three-profile and four-profile models. In terms of class distinguishability, the three-profile model clearly distinguished three groups with different characteristic patterns, demonstrating a concise structure and strong theoretical interpretability. In contrast, in the four-profile model, Class 2 and Class 3 exhibited highly similar score patterns across items, lacking independent substantive discriminative meaning. Regarding changes in fit indices, the decreases in AIC, BIC, and aBIC for the four-profile model had notably slowed down. For example, the decrease in BIC from the three-profile to the four-profile model was only 230.913, far smaller than the decrease of 630.525 from the two-profile to the three-profile model. In terms of model stability, the three-profile model had relatively balanced class proportions (19.53, 43.65, 36.82%), whereas in the four-profile model, Class 2 accounted for only 9.21%—a small class size susceptible to sampling fluctuations, raising concerns about cross-sample stability and overfitting risks. This judgment was further corroborated by the results of mediation analyses. We conducted an additional multi-categorical mediation analysis based on the four-profile model. In this model, Class 1 represented the “low-level” group with generally low scores across all items, Class 2 represented the “moderate-level” group, Class 3 represented the “relatively high-level” group, and Class 4 represented the highest-scoring group. With Class 1 as the reference group, the results showed that the mediating effect of Class 2 relative to Class 1 was significant, while that of Class 3 relative to Class 1 was not. This violated the expected monotonic trend of class-level effects, suggesting that Class 3 may be a statistically overfitted artifact rather than a substantively meaningful psychological class. Based on a comprehensive consideration of fit statistics, class distinctiveness, model stability, and theoretical meaningfulness, this study ultimately selected the three-profile model as the optimal latent profile model.

**Figure 1:**
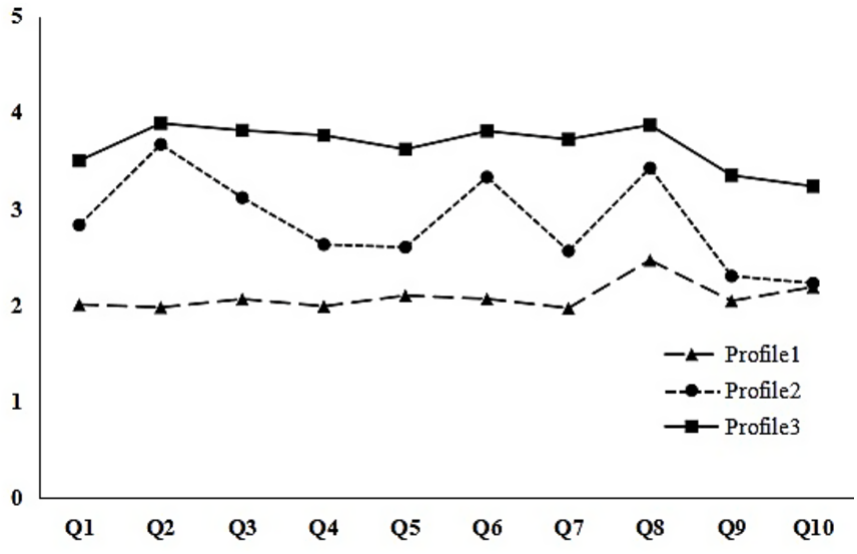
The three profiles of academic delay of gratification by latent profile analysis.

Based on the characteristics of the model (Figure 1), there are three potential profiles of ADOG among the sample of college students. Among them, the first class has 140 people, accounting for 19.53%; the second class has 313 people, accounting for 43.65%; and the third class has 264 people, accounting for 36.82%. Based on the model characteristics and the scores of the ADOG questionnaire, the 3-profile model is named as follows: The first class has the lowest score of ADOG, so it is named “low ADOG”; the second class has a medium score of ADOG, so it is named “medium ADOG”; the third class has the highest score of ADOG, so it is named “high ADOG”.

### 4.3 Profile differences in academic involution and academic self-efficacy

According to the data reported in Table 3, the scores of academic involution and academic self-efficacy across different academic delay of gratification (ADOG) latent profile types showed a consistent order: high ADOG profile > medium ADOG profile > low ADOG profile. Analysis of variance (ANOVA) was adopted to examine the differences in academic involution and academic self-efficacy among the three ADOG profiles. The high ADOG profile group exhibited the highest scores in both academic involution and academic self-efficacy, while the low ADOG profile group showed the lowest scores in these two variables. The LSD post-hoc test revealed that the academic involution score for high ADOG (*M* = 3.50) was significantly higher than the other two types (*p* < 0.001). The academic involution scores for medium ADOG (*M* = 3.15) and low ADOG (*M* = 2.99) were both moderately high, and there was a significant difference between the two groups (*p* < 0.001). The three profiles also showed significant differences in academic self-efficacy, with the high ADOG group scoring the highest (*M* = 3.76), the medium ADOG group in the middle (*M* = 3.50), and the low ADOG group the lowest (*M* = 3.39; Table 3).

**Table 3:** Profile differences in academic involution and academic self-efficacy (M ± SD).

Variables	Profile 1 (*N* = 120)	Profile 2 (*N* = 238)	Profile 3 (*N* = 218)	*F*	*η^2^*	Post-hoc
Academic involution	2.99 ± 0.82	3.15 ± 0.70	3.50 ± 0.73	26.79^***^	0.07	1 < 2 < 3
Academic self-efficacy	3.39 ± 0.80	3.49 ± 0.62	3.76 ± 0.73	15.98^***^	0.04	1 < 2 < 3

***p < 0.001.

### 4.4 Mediated effects of academic self-efficacy

We analyzed the mediating effect of academic self-efficacy using Model 4 in the PROCESS v4.0 macro for SPSS, while controlling for variables including gender, education level, school type, and major type (Hayes, 2018). Specifically, a simple mediation analysis was adopted for the variable-centered perspective of mediating effect analysis, whereas a multi-categorical mediation analysis was used for the person-centered perspective.

### 4.5 Mediation analyses: variable-centered approach

**Figure 2:**
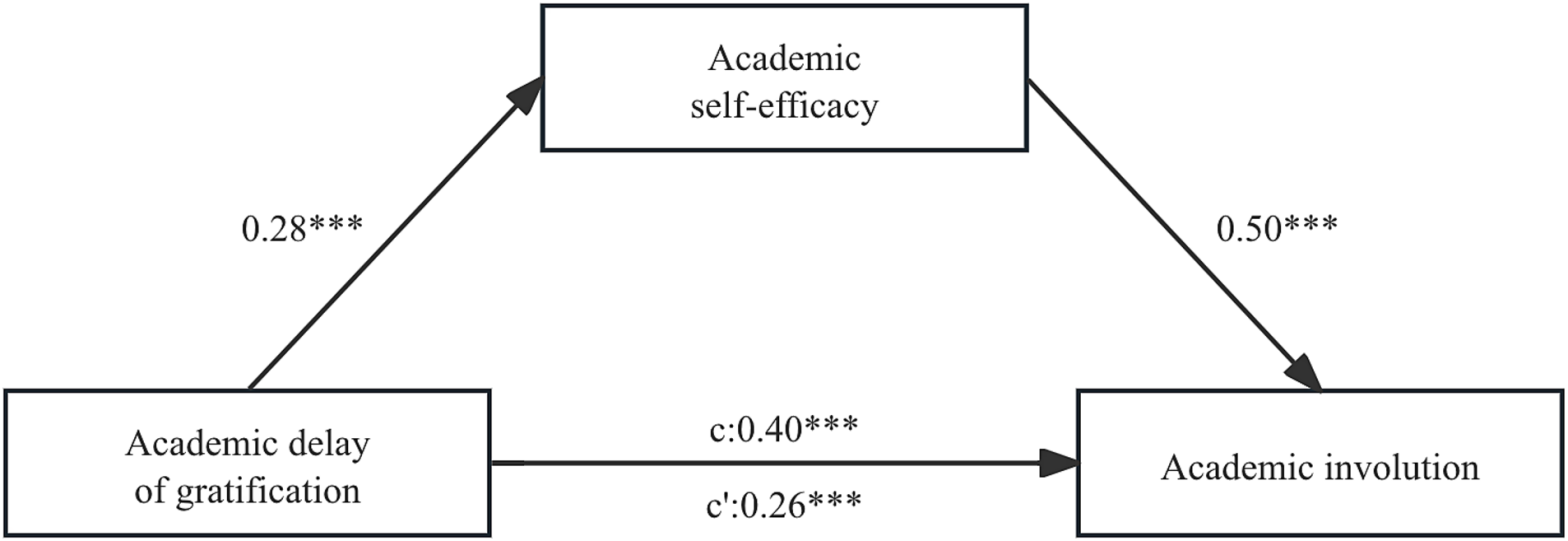
Overall mediation analysis model (***p < 0.001).

**Figure 3:**
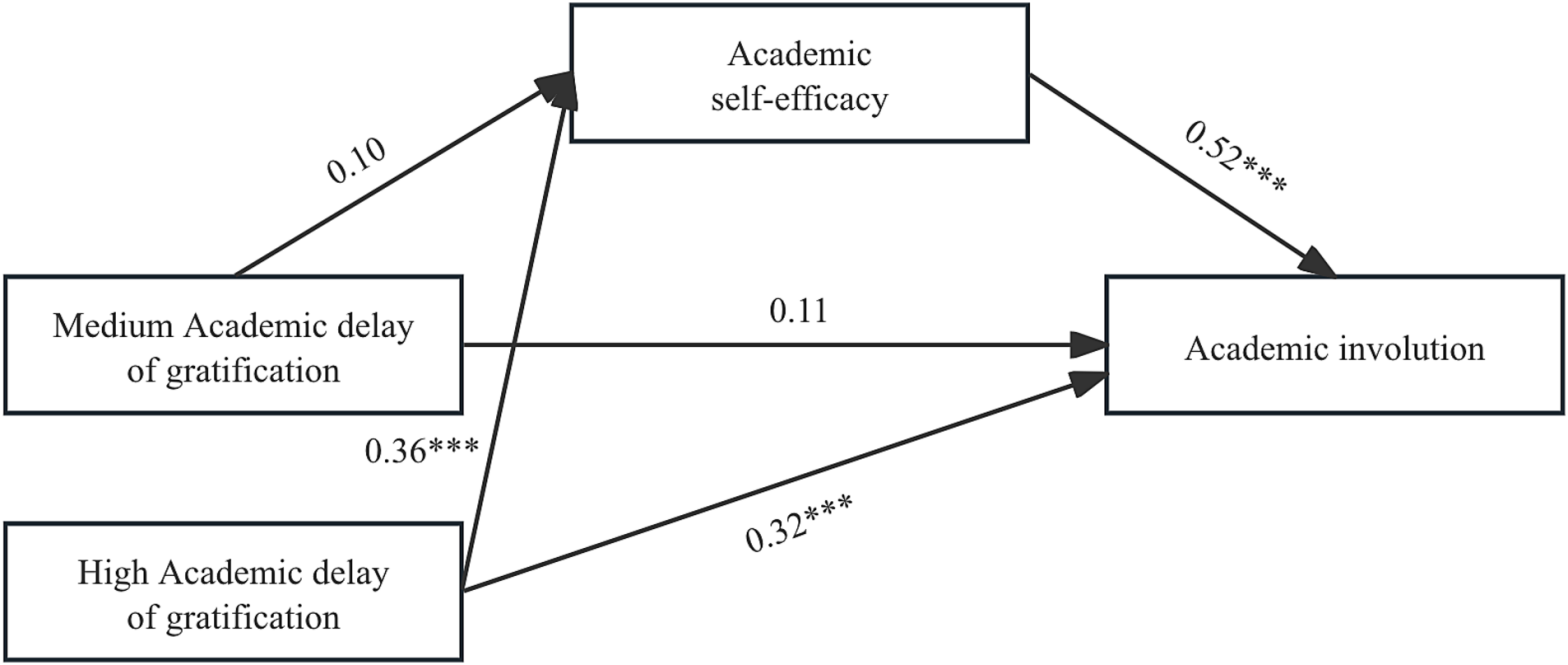
Relative mediation analysis model (***p < 0.001).

To investigate the mediating role of academic self-efficacy between ADOG and academic involution, this study utilized Model 4 in the PROCESS program of SPSS software for analysis. Figure 2 displays the outcomes of the path test for the overall mediating model. The results indicated that ADOG had a significant positive predictive effect on academic self-efficacy (*β* = 0.28, *p* < 0.001) and a significant positive total effect on academic involution (*c* = 0.40, *p* < 0.001). In addition, academic self-efficacy was found to significantly and positively predict academic involution (*β* = 0.50, *p* < 0.001). After including academic self-efficacy in the model, ADOG still had a significant positive direct effect on academic involution (c’ = 0.26, *p* < 0.001), confirming the partial mediating role of academic self-efficacy between ADOG and academic involution. To further explore the differences in the mediating effect under different levels of ADOG, this study subsequently conducted a relative mediation analysis (Figure 3).

### 4.6 Mediation analyses: person-centered approach

To represent the three ADOG profiles, we generated two dummy variables (medium ADOG and high ADOG) through indicator coding, taking the low ADOG profile as the reference group. The results of the relative mediation analysis are presented in Table 4. Data indicate that for medium ADOG (D1), the 95% confidence interval of academic self-efficacy’s relative indirect effect is [−0.03, 0.13], which includes 0, confirming no statistically significant indirect effect of ADOG on academic involution via academic self-efficacy at this level. The 95% confidence interval of the relative direct effect in the medium ADOG group is [−0.02, 0.24], also including 0, suggesting that the direct effect of ADOG on academic involution at this level is not statistically significant either. In the high ADOG group (D2), the 95% confidence interval of academic self-efficacy’s relative indirect effect is [0.11, 0.27], which does not include 0, indicating that at this level, ADOG indirectly and positively predicts academic involution through academic self-efficacy. The 95% confidence interval of the relative direct effect in the high ADOG group is [0.19, 0.46], which does not include 0, suggesting that ADOG significantly and positively predicts academic involution. Comparing the three profiles of ADOG, it can be seen that compared with low and medium ADOG, when individuals are at the “high ADOG” level, three effects are found to be more significant and exhibit positive predictive features. These effects include the indirect effect of ADOG on academic involution via academic self-efficacy, the direct effect of ADOG on academic involution, and the total effect. This indicates that within this latent profile group, ADOG positively predicts academic involution by enhancing academic self-efficacy.

**Table 4:** Relative effects of academic self-efficacy on academic involution across different profiles of ADOG.

Effect type	Variables	Effect	SE	95% CI	
				Lower	Upper
Relative indirect effects^#^	D1	0.05	0.04	−0.03	0.13
	D2	0.19	0.04	0.11	0.27
Relative direct effects^#^	D1	0.11	0.07	−0.02	0.24
	D2	0.32	0.07	0.19	0.46
Relative total effects^#^	D1	0.16	0.08	0.02	0.31
	D2	0.52	0.08	0.36	0.66

#“low ADOG” is the reference; D1 and D2 represent dummy variables for “medium ADOG” and “high ADOG,” respectively; controlled for gender, education level, majors. CI, Confidence interval; SE, Standard error.

## 5 Discussion

### 5.1 Relationship between ADOG and academic involution

Our study finds that higher levels of ADOG among college students are associated with greater academic involution, thereby supporting Hypothesis 1. This finding aligns with the energy model of self-control, which posits that self-control energy is a limited resource (Baumeister et al., 2007). College students with high ADOG are better able to sustain long-term efforts for future academic rewards. Consequently, when facing academic competition, they are more inclined to continuously expend self-control energy to resist immediate gratification and adhere to long-term academic goals. However, when self-control energy becomes excessively depleted, these students may struggle to maintain high-intensity engagement and competitive states, leading to diminished efficiency or even ineffective investment. Furthermore, ADOG may exacerbate negative behaviors in high-pressure environments. Students who are adept at delaying gratification tend to hold higher expectations for goal attainment, which may prompt them to continually extend study time and intensify their efforts under competitive pressure, ultimately falling into a state of excessive competition. This pattern is consistent with the findings of Zhou et al. (2022) on the interplay between academic involution and academic adaptation.

It can be seen that ADOG has a dual role in a competitive context. From the perspective of self-regulated learning theory (Zimmerman, 2000), ADOG is essentially a dynamic self-regulatory process. When self-regulation is successful, students can effectively implement ADOG, accumulate more learning resources, and maintain efficient learning engagement. For instance, Chen and Woody (2026) found that individuals with moderate levels of ADOG tend to set long-term goals, adopt effective learning strategies, and achieve superior academic performance. Conversely, when self-regulation fails, two different types of negative outcomes may emerge. If the level of ADOG is too low, individuals struggle to resist immediate temptations, which may lead to academic procrastination, a result that aligns with Zhao et al. (2021). On the other hand, when ADOG is overused, individuals continuously deplete their cognitive resources under sustained high-intensity competitive pressure without adequate recovery, thereby falling into self-depletion through inefficient “involution” (Liu et al., 2022). Notably, the negative effects of ADOG are not exclusive to the Chinese educational context. In the highly competitive educational environment of South Korea, students’ overinvestment in long-term academic rewards leads to excessive academic pressure and emotional exhaustion (Ermolenko and Siddikova, 2025). Similar psychological mechanisms have also been observed in Singapore. For instance, Puah et al. (2024) found that high-achieving university students, driven by the continuous pursuit of better performance, face a significant risk of burnout. Thus, while colleges and universities cultivate students’ ability to delay gratification, they should also attach importance to guiding them to establish scientific goal concepts and competitive cognition to avoid high investment turning into low-efficiency self-depletion.

### 5.2 ADOG profiles

Using LPA, we identified three distinct ADOG profiles among college students: low ADOG (19.53%), medium ADOG (43.65%), and high ADOG (36.82%), thereby supporting Hypothesis 3. This heterogeneity is consistent with previous research. As an important manifestation of self-regulation ability, the classification pattern of ADOG partially aligns with broader findings on academic self-regulation. For example, Chen and Yeung (2025) found that students’ self-regulated learning patterns exist at three levels—limited, moderate, and superior—and that students with stronger self-regulation abilities demonstrate significantly greater learning engagement. Furthermore, the characteristics of the “high ADOG” group identified in this study resemble the psychological and behavioral profiles of “competent regulators” described by Lin J. W. et al. (2023). Specifically, compared with their low and medium ADOG peers, college students exhibiting high ADOG show stronger self-control and greater persistence when facing academic pressure.

The results also show significant differences across the three ADOG profiles in both academic involution and academic self-efficacy, further supporting Hypothesis 3. Students with high ADOG exhibited the highest levels of academic involution, followed by those with medium and low ADOG. This finding suggests that high ADOG is closely associated with greater academic engagement, partially confirming previous research. For instance, Duckworth et al. (2019) reported that self-control positively predicts college students’ academic achievement. The present study further reveals that in competitive academic environments, ADOG may specifically manifest as sustained, high-intensity engagement, thereby intensifying academic involution. Additionally, students with high ADOG not only reported significantly higher levels of academic involution compared to their medium and low ADOG counterparts but also demonstrated significantly stronger academic self-efficacy. This indicates that higher ADOG is associated with greater confidence in one’s learning abilities, consistent with Bembenutty (2022). These profile differences provide a foundation for further exploring the relationships among ADOG, academic self-efficacy, and academic involution.

### 5.3 Mediation of academic self-efficacy

The variable-centered path analysis reveals that academic self-efficacy partially mediates the relationship between ADOG and academic involution, thereby supporting Hypothesis 4. As a self-regulatory behavior, ADOG directly strengthens an individual’s belief in their ability to meet academic requirements, that is, it significantly enhances academic self-efficacy (Freire et al., 2020). Individuals with higher academic self-efficacy form positive expectations about their capacity to overcome academic challenges. This confidence translates into stronger competitive motivation, prompting them to devote more time and effort to consolidating their advantages, thereby exacerbating academic involution (Locke and Latham, 2002; Ein-Gar and Steinhart, 2017). Thus, academic self-efficacy serves as a key mediator between ADOG and academic involution.

From this person-centered perspective, we identified three ADOG profiles among college students. Furthermore, we examined whether academic self-efficacy mediates the relationship between academic involution and each ADOG profile. The results show that, compared to the low ADOG groups, the high ADOG group exhibits a distinct pattern. Only among individuals in the high ADOG state does ADOG not only directly predict academic involution but also indirectly relate to it through enhanced academic self-efficacy.

Our investigation further confirms the Social Cognitive Theory’s assertion that self-efficacy is associated with one’s choices regarding activities, efforts, and persistence (Bandura, 1977). According to SCT, individuals with high self-efficacy demonstrate a higher propensity for perceiving academic challenges as achievable goals rather than insurmountable obstacles, and this cognition will be transformed into a stronger competitive motivation (Locke and Latham, 2002). Additionally, SCT emphasizes the function of individual heterogeneity in the interaction process, which can also explain the differences in the mediating effects among different ADOG profiles in this study. For the high ADOG, their long-term and stable self-regulatory behaviors will continuously provide positive feedback to self-efficacy, making the mediating function of self-efficacy more significant. Therefore, by identifying students of different profiles of ADOG, implementing a group-based intervention model is beneficial for precisely alleviating the involution pressure faced by different student groups and achieving positive guidance of academic competition.

### 5.4 Research implications

This study employed LPA to explore the latent profiles of ADOG among college students, as well as differences in academic self-efficacy and academic involution across these profiles. The empirical findings elucidate the mechanisms linking ADOG, academic self-efficacy, and academic involution, thereby contributing a theoretical framework for future research in this area. Furthermore, by using LPA to identify heterogeneous characteristics of ADOG across different groups, this study moves beyond previous research that focused on unidimensional linear effects. The results further confirm that significant differences exist in the mediated pathway from ADOG to academic involution via academic self-efficacy. Specifically, this indirect effect is significant only among individuals with a high level of ADOG.

On a practical level, based on empirical findings regarding group heterogeneity and threshold effects in ADOG, this study provides actionable strategies for precise intervention in academic involution, rather than merely clarifying theoretical associations. On the one hand, the study identifies the key target population for intervention: educators should prioritize college students with high ADOG. For this group, intervention programs can be designed to guide rational competition. On the other hand, the study offers directions for academic support among students with low and moderate ADOG. By helping these students enhance their ability to delay gratification, we can prevent the widening of academic disparities driven by self-efficacy, thereby indirectly reducing unproductive competition at the group level caused by ability stratification.

Based on the research findings, we propose the following recommendations. First, educators should help students manage their time reasonably to avoid distraction or excessive engagement driven by the pursuit of academic advantage. For contemporary college students, competing demands and easy access to distractions make time management challenging. Research indicates that academic time planning is positively associated with higher GPA, and the self-regulatory process of task planning also affects academic outcomes (Thibodeaux et al., 2017). Furthermore, Xia et al. (2023) noted that excessive video watching and gaming tend to distract attention, thereby influencing ADOG. Therefore, helping students master time management strategies and formulate feasible academic plans is practically important. Second, this study found that both ADOG and academic self-efficacy represent important forms of self-control and self-management, playing critical roles in achieving academic goals. Students should proactively recognize and employ metacognitive strategies, adopt effective methods, seek resources, and continuously engage in self-adjustment to enhance their academic performance. Moreover, policymakers may guide universities in optimizing academic evaluation systems by reducing single-indicator, grade-oriented assessments and incorporating greater consideration of learning processes and comprehensive abilities, thereby fostering a healthier academic competition environment at the institutional level.

## 6 Limitations and future study

Although this study holds theoretical and practical value, several limitations should be acknowledged. First, the cross-sectional design limits our ability to capture dynamic processes over time and to infer causal relationships between ADOG and academic involution. Meanwhile, the questionnaire-based survey inevitably introduces self-report bias. Respondents may be subject to social desirability effects, leading them to report academic behaviors that conform to societal expectations while masking their true perspectives. Additionally, the use of convenience sampling may limit the generalizability of our findings to the broader population of Chinese college students. Future research should employ probability sampling methods (e.g., stratified random sampling) to replicate and extend this study across diverse institutional and regional contexts. Accordingly, multi-source data and longitudinal designs should be considered to provide more rigorous causal evidence regarding the relationships and mediating mechanisms among the variables.

Second, the data for this study were drawn from a sample of Chinese college students, and the manifestation and causes of academic involution may be deeply influenced by the local social and cultural context. In the Chinese cultural context, academic involution is not merely individual competition but also a matter of family honor. As Chui and Wong (2017) noted, Chinese parents often hold high expectations for their children’s educational attainment, turning academic competition into a means of preserving family social status, which reinforces the persistence of involution. This cultural specificity, while enhancing the authenticity of the research context, limits the generalizability of the findings to other cultural and educational environments. The manifestations, triggers, and adaptive outcomes of academic involution may differ considerably depending on social norms, educational systems, and values. Therefore, future research should test these findings across cross-cultural samples to assess the external validity and cross-contextual stability of our conclusions.

Finally, academic involution is a complex social phenomenon whose manifestations and causes may extend beyond the individual psychological level. Although this study identified the mediating role of academic self-efficacy, the development and persistence of academic involution are likely shaped by other significant variables, including achievement goal orientation, stress coping strategies, and perceived parental educational expectations. Future research should construct a more integrated theoretical model that incorporates multi-level variables at the individual, family, and school levels to systematically elucidate the mechanisms linking ADOG to academic involution.

## 7 Conclusion

This investigation reveals the heterogeneity of ADOG among college students, uncovers the intrinsic mechanisms linking ADOG, academic involution, and academic self-efficacy through both variable-centered and person-centered approaches, and provides a preliminary basis for guiding college students in coping with academic competition. Four main conclusions are drawn from the study: (1) ADOG significantly and positively predicts academic involution; (2) Latent profile analysis identified three distinct profiles of ADOG, namely low, medium, and high, with significant differences across profiles in both academic self-efficacy and academic involution; (3) Academic self-efficacy partially mediates the positive relationship between ADOG and academic involution; (4) Multi-categorical mediation analysis further revealed significant group heterogeneity in this mediating mechanism. Specifically, the mediating role of academic self-efficacy is significant within the high ADOG profile relative to the low ADOG reference group.

## Data Availability

The raw data supporting the conclusions of this article will be made available by the authors, without undue reservation.
